# Willingness to pay for a second pair of near-vision glasses: a cross-sectional study in a rural North Indian population

**DOI:** 10.1186/s12889-025-22278-2

**Published:** 2025-04-22

**Authors:** Shalinder Sabherwal, Javed Nayab, Atanu Mazumdar, Nam Thaker, Mohd Javed, Rakhi Nathawat, Andrew Bastawrous

**Affiliations:** 1 Dr. Shroff’s Charity Eye Hospital, Delhi, India; 2 International Centre for Eye Health, Department of Clinical Research, Faculty of Infectious and Tropical Diseases, London School of Hygiene & Tropical Medicine, London, UK; 3 Peek Vision, Berkhamsted, UK

**Keywords:** Presbyopia, Near-vision glasses, Willingness to pay

## Abstract

**Purpose:**

There is an enormous unmet need for near vision correction with glasses. The cost and lack of felt need are important barriers. This study, which was conducted among a rural population of northern India, was designed to assess whether the short-term use of a pair of near-vision glasses can increase the desirability for individuals to procure subsequent pairs and to further assess the willingness to pay thresholds.

**Methods:**

This study followed a quasi-experimental design. Uncorrected presbyopes were given near vision glasses at their doorstep, to carry out their chosen near work task for half- an- hour (this use of glasses was referred to as ‘experience’ for the purpose of this study). They were then referred to nearby vision centres to procure glasses. This ‘experience’ given was used as a proxy for having used the first pair. At the vision centre, glasses were offered at no cost, for Indian Rupees 75 (US$0.90) and for Indian Rupees 100 (US$1.20) in the first, second and third phases of the study, respectively. The usual price at which near-vision glasses were otherwise available in the region was Indian Rupees150 (US$1.8). The uptake of glasses after having received the near correction experience was tracked via the Peek Vision platform.

**Results:**

The most preferred chosen near work task by the study participants were stitching, after threading the needle and using a mobile phone. The uptake of near-vision glasses from the vision centre after providing the desired experience was 81.4% (835/1,026), 48.3% (699/1,446) and 29.2% (93/318) when the glasses were provided free of cost, at $0.90 and at $1.20 respectively. The difference between these three phases was statistically significant (*p* < 0.001). Uptake was found to be increase with need for increasing lens power (*p* < 0.01) and especially among those who reported the ‘experience’ as ‘very good’ or ‘excellent’(*p* < 0.001). Uptake decreased with increasing age (*p* < 0.01). Differences in uptake between sexes and between those with or without the availability of a mode of transport in their household were not found to be significant.

**Conclusion:**

Having experience with the first pair of near-vision glasses can increase desirability of procuring subsequent pairs. Offering the second pair at a reduced price can increase the uptake substantially in this setting, suggesting that active outreach to correct near vision in tandem with accessible and affordable marketplaces for reading glasses could provide a viable solution to scale near vision correction.

**Supplementary Information:**

The online version contains supplementary material available at 10.1186/s12889-025-22278-2.

## Introduction

Presbyopia, a common age-related condition characterized by a decreased ability to focus on near objects, affects a significant portion of the global population. With an estimated 1.8 billion individuals worldwide experiencing this condition, it represents a substantial public health concern [1]. Among these, an estimated 826 million individuals currently have untreated presbyopia, highlighting the urgent need for effective interventions [2]. Few studies have investigated the impact of uncorrected presbyopia on quality of life. A study by Berdahl et. al. revealed that people with presbyopia often report a 22% decrease in quality of life as compared to the controls [3]. Many experience difficulties in performing near-vision tasks, leading to increased dependency on caregivers [3]. The annual global productivity loss due to uncorrected presbyopia in people under 65 years of age was estimated to be US$25 billion in 2011 [3]. In the PROSPER trial carried out among tea-pickers in India, the increase in productivity in the group provided near-vision glasses was found to be approximately 22% [4], whereas in the THRIVE trial conducted among people in occupations involving intense near work, there was a 33% increase in income for those who were provided near-vision glasses [5]. In another study by Wubben et al., 84% of participants stated that glasses could improve their ability to earn. [6] Thus addressing untreated presbyopia aligns with the United Nation’s Sustainable Development Goals (SDGs)- SDG1 (No Poverty), SDG 2 (Zero Hunger) and SDG8 (decent work and economic growth) [7].

The prevalence of presbyopia in India has been reported to vary between 45 and 61% in people over 40 years of age in population-based studies [8–10]. The coverage of presbyopia correction has been reported to vary between 32 and 43% [9, 10]. Although, it has been reported to be as low as 11% in some communities [8]. The main barriers to the uptake of near-vision glasses reported are associated costs and a lack of perceived need by the individual [11–14]. In a study by Marmamula et al., cost has also been reported as a reason for discontinuation of using glasses once they are lost or broken [15].

On average, a person may need to replace their near-vision glasses every two years, resulting in a potential need for ten pairs over a 20-year period. Factors such as the severity of presbyopia, usage patterns, and lens quality can influence the lifespan of glasses with some requiring more frequent replacements than others do. Understanding the factors influencing an individual's willingness to pay for subsequent pairs of glasses is crucial for estimating the lifetime cost of providing near-vision correction to those with unmet needs. There are studies that have assessed the willingness to pay for near-vision glasses [16] and Guan et al. have reported an increase in the uptake of vision care services after being provided near-vision glasses with subsidies [17]. However, very few studies have explored the effect of the use of near-vision glasses on willingness to pay for subsequent pairs. One such study by Laviers et al. reported an increase in willingness to pay after usage, however, only 187 people were included in that analysis [14]. Another study reported a marginal increase in willingness to pay for glasses among those shown educational videos [18].

From a developmental perspective, governments and funding agencies like international non-governmental organizations and corporations supporting healthcare, can be a source for providing free near-vision glasses and their delivery, but whether providing a first pair of glasses free of cost can create a willingness to pay for consecutive pairs and thus, reduce the amount of resources required in the long run needs to be studied.

The research question for this study was- whether using the first pair of near-vision glasses would increase the willingness to travel and pay for subsequent pairs of reading glasses among those who needed them. As the need for a change in near-vision glasses may manifest after 2–3 years, we used the initial experience of using near-vision correction at their doorstep as a proxy for having had a first pair of near-vision glasses to assess whether the experience led to a change in behaviour related to accessing readers through the typical delivery channels in that region at varying prices.

This study was embedded in an ongoing screening and referral program delivered in partnership with Peek Vision [19]. In this program, screeners travel door-to-door in the villages and screen all individuals of the household aged 5 and above for vision and any other obvious eye conditions. Those found with a need for intervention or further evaluation are referred to the nearby vision centres. Six months of programmatic data prior to the start of this study revealed 4,199 people had been referred with near vision related issues, only 1,285 (30.6%) of those had attended at the vision centre to procure glasses.

## Methods

In this study, participants were provided with experience of using near-vision glasses at their doorstep, which was used as a proxy for the use of the first pair of near-vision glasses. Next it was assessed whether they would travel to a nearby vision centre to procure a pair of near-vision glasses that were provided free or at two price-points in different phases of the study.

**Fig. 1:**
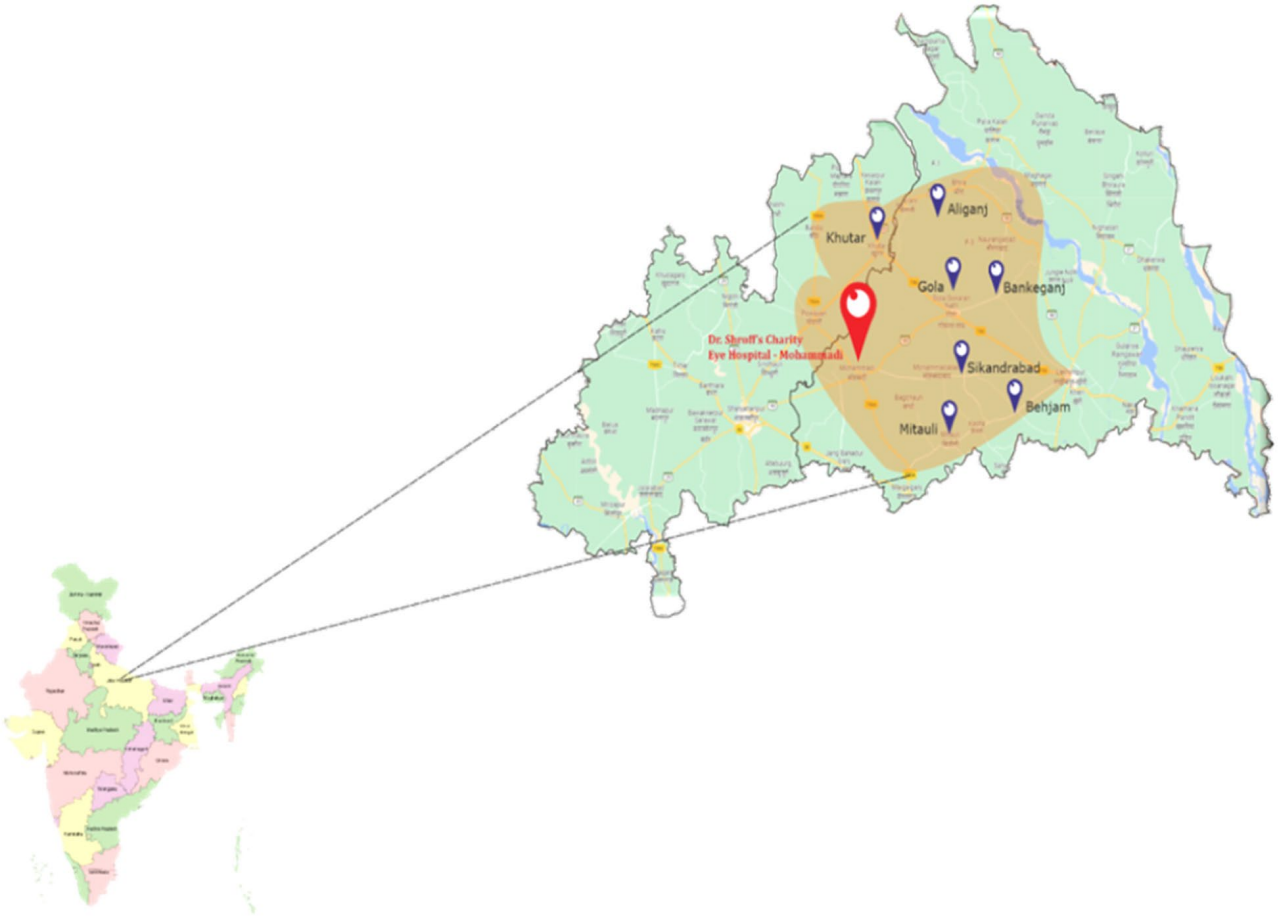
Location of ongoing door-to-door screening program using Peek

This study was conducted from April 2023 to February 2024. This study employed a quasi-experimental design and was nested in an ongoing door-to-door screening program conducted in the villages belonging to the state of Uttar Pradesh, located in northern India (Fig. 1). In this program all household members aged 5 and above are screened for vison and any obvious eye conditions. This screening program operates across two districts—Shahjahanpur and Lakhimpur Kheri, within the catchment area of our surgical centre, which is situated in Uttar Pradesh, a state in northern India. More than 90% of the population of the districts included in this study is rural [20].

The existing program connects screeners, vision centres and the local eye hospital through Peek’s data intelligence platform, which enables smartphone-based screening and referral along the patient pathway. Peek software has the ability to track in real-time how people screened move through the system. The study screeners received additional training over a period of 2 weeks for this study. Training conducted by a clinical optometrist included capturing distance visual acuity via Peek Acuity [21], recording near visual acuity using standard near vision chart at 40 cm and performing subjective refraction for near correction in those with distance visual acuity of better than 20/40 in both eyes and a near vision impairment, which was defined as both eyes having near vision less than N6 at 40 cm. They were also trained to collect screening data on the Peek platform and to use open data kit (ODK) [22] software to collect consent and data related to the specific near vision experience provided. Hands-on training was provided on patients in the hospital under supervision. Twenty patients needing near vision correction were examined by each of the screeners and the results were compared with those of the masked clinical optometrist. The study was started only when an agreement of more than 70% was achieved between the screeners and the optometrist on the prescription provided to the patient for near vision.

### Inclusion and exclusion criteria for the study participants

Inclusion criteria:

- Individuals who’s near visual acuity did not improve to N6 or by two lines with near vision correction

Exclusion criteria:

- Individuals who’s near visual acuity did not improve to N6 or by two lines with near vision correction
- Individuals with distance visual acuity more than 20/40 but found with any obvious eye condition
- Individuals who had previously been treated with glasses for near vision correction
- Individuals who refused to provide consent for the study

The eligible individuals were provided accurate near vision glasses for 30 min to perform their chosen near vision activity, referred to as ‘experience’ for the purpose of this study. The modalities for providing experience at the doorstep were chosen after 50 men and women were asked before the study, regarding the near vision activities they most commonly perform or experience difficulties in. The activities finalised for the study were – threading the needle for stitching (supplementary file-photo1), cleaning grains by removing gravel (Supplementary file-photo2) reading a newspaper and using their mobile phones (nonsmart) (Supplementary file-photo-3). Kits were made for each of these activities (except for using mobile phones) and the teams carried these kits with them.

**Fig. 2:**
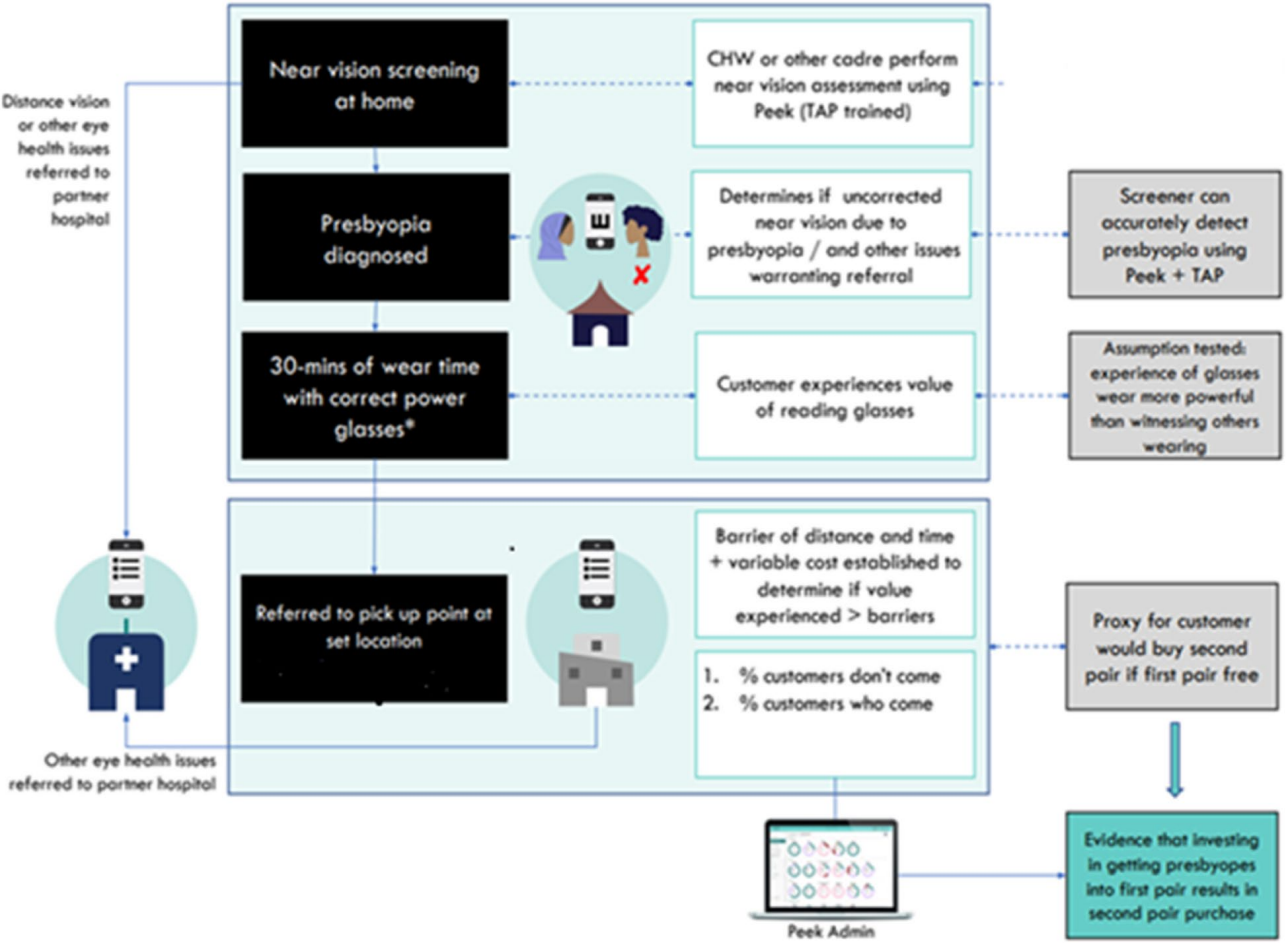
Figure describing the process flow of the study

Figure 2 depicts the process flow. Distance visual acuity was tested followed by near visual acuity for those with uncorrected distance visual acuity of better than 20/40 in both eyes. Individuals with distance visual acuity less than 20/40 in either eye or any other obvious eye conditions were excluded from the study and were referred directly to vision centre. Those who were eligible for the study after near vision testing were provided a pair of near-vision glasses with which they could see N6 on the near vision chart or their near vision improved by at least 2 lines. This was followed by providing near vision ‘experience’. The eligible participants were asked for the near activity that they perform most often before using a particular kit for the experience. They were provided with an accurate pair of glasses for a period of 30 min to perform the selected activity. At the end of that period, the participants were asked to rate their experience as – not good, good, very good, or excellent.

One team comprised one study-screener trained to carry out near vision related data collection, one screener of the ongoing door-to-door screening program and a field coordinator. The preexisting door-to-door screener was responsible for conducting eye screening of all household members aged 5 and older. Those above 35 years of age and with uncorrected distance vision of more than 20/40 in both eyes were referred to the study screener. The study screener was then responsible for checking near vision, testing for near correction and providing the near vision ‘experience’ with near vision glasses to the eligible participants. Having a separate study screener in a team ensured that the routine ongoing screening program proceeded without any delays. The field coordinator was responsible for facilitating screening in the villages by engaging with key stakeholders like elected village head, senior schoolteachers and government appointed community health workers. Three teams of three people each were created. One program manager was responsible for monitoring the screening to ensure compliance with the protocol.

A total of 4,093 villages were available to sample in the two districts within the catchment area of our hospital. As this study was nested within an ongoing screening program, only 544 villages located within the program area were included in randomization for inclusion in the study. Villages situated within 3 km distance of those included in the previous phase were excluded from the subsequent phases. Phase 1 included providing ‘experience’ to the eligible individuals and referring them later to the nearby vision centre for collecting glasses. In this first phase glasses at the vision centre were offered free of cost to study the impact of distance, travel and other indirect costs on uptake.

It was decided to first meet the sample size requirements of the first phase before implementing the subsequent phases. The decision to undertake the next phases was made dependent on the uptake observed in the first phase. We decided to proceed to the next phases of offering glasses at a price instead of free, only if the adherence in the first phase was at least 20 percentage points above the programmatic data available prior to the study which revealed an uptake of 30.6%.

In the subsequent phases 2 and 3, glasses were offered at Indian Rupees 75 (US$0.90) and at Indian Rupees 100 (US$1.20) respectively at the vision centre to the study participants referred from the villages. The usual price at which near-vision glasses were otherwise available in the region was Indian Rupees 150 (US$1.8). Phases 2 and 3 were carried out to study the willingness to pay and thus additional barrier of direct cost in this rural catchment. All three phases were carried out in different villages that were in the same study region but distant from each other.

#### Sample size

The sample size for this study was calculated to address the following research questions:

- The proportion of presbyopic individuals who experienced improved vision using glasses, are willing to acquire the glasses on their own and how does willingness change when glasses are provided for free versus when they are offered at a cost?
- For a significance level (α) of 0.05, and margin of error (d) of 0.05, assuming maximum variance, the statistical sample size calculated was 385. Given that the study employed multilevel sampling, a design effect of 2 was applied, resulting in a minimum sample size of 770, rounded to 800 per group.
- For the second and third phases, where we introduced a payment instead of free glasses, we decided that we would work with a 95% confidence interval of ± 10%, require a minimum sample size of 194, (rounded to 200) and would proceed to a sample size of 800 with a 95% confidence interval of + 5%, only if there is a trend of uptake greater than the routine programmatic uptake.
- Variables for which data were collected for all three phases were- age of the participants, sex of the participants, distance of the village from the vision centre, type of near vision ‘experience’ provided at the doorstep, reporting of experience as not so good, good, very good or excellent by participants, power of the near vision glasses provided at the doorstep and ownership of personal mode of transportation in the household or dependence on public transportation for travelling to the vision centre.

#### Data analysis

Data were analysed to determine the number of participants who received near vision spectacles after being advised. Statistical significance between different groups was assessed using the chi-square test, with P-values calculated to determine the level of significance. Multivariable logistic regression was used to assess the associations between various factors and the uptake of spectacles. The statistical analysis was performed using R version 4.4.2. Charts were created using MS Excel.

#### Ethical considerations

Informed consent was obtained from all participants, and the study adhered to ethical standards as per the Declaration of Helsinki. The confidentiality of the participants information was maintained throughout the study. Individual consent was obtained to capture and use photos of the participants. Ethical approval for this study was received from the IRB board of Dr Shroff’s Charity Eye Hospital (IRB/2023/MAR/146).

## Results

A total of 53,951 individuals belonging to 65 villages were screened during the study period. Of these 19,312 were above 35 years of age and 3,312 were found to be eligible for inclusion in this study. A total of 3,141 (94.8%) individuals consented for the study and all of them were referred to the vision centres across the three phases. Among these, 351 were excluded because of data inconsistencies. A total of 2,790 participants were included in the analysis- 1026 in phase 1, 1446 in phase 2 and 318 in phases 3. The participants were offered glasses at no cost in phase 1, at US$0.90 in phase 2 at US$1.20 in phase 3. There were 1,507 women and 1,283 men in the three groups combined. The average age of the participants was 45 years (45.19 ± 7.05).

**Fig. 3:**
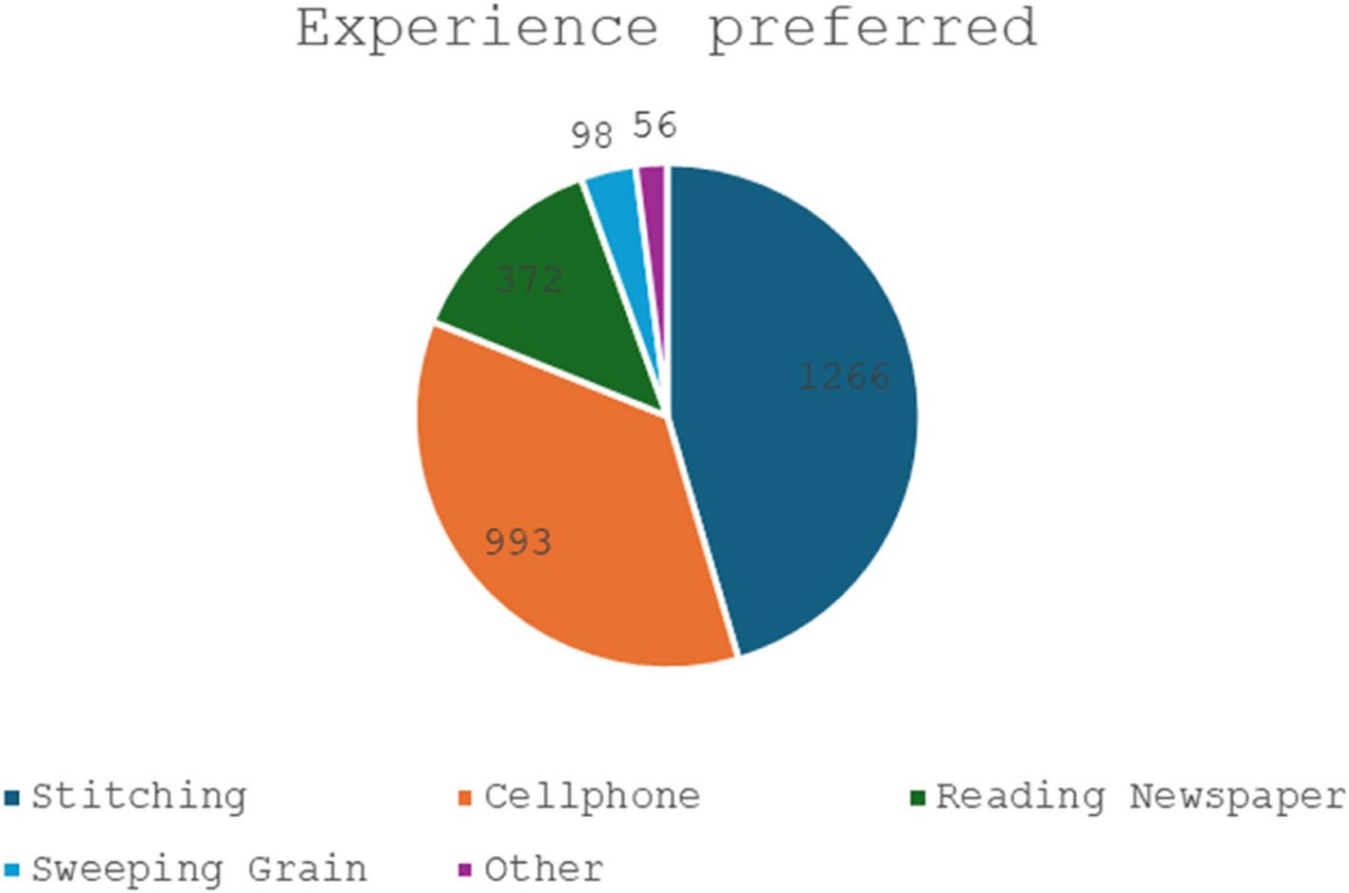
Distribution of near vision experiences preferred by study participants

In all, data for the type of experience given were available for 2,785 participants. Stitching after threading the needle was the most preferred experience (Fig. 3).

The uptake of glasses with different near vision ‘experiences’ is detailed in Table 1. The difference between the groups was not statistically significant. Maximum uptake was observed among those who chose the sweeping-grain activity (70.4%) followed by mobile phone handling, stitching and newspaper reading. The uptake rates were found to be significantly varying across groups associated with different types of experiences (*p* = 0.048).

**Table 1:** Uptake of glasses associated with different ‘experiences’

Type of experience	Referred to vison centre	Uptake at vision centre *n*, (%)	p-value
Stitching	1266	728, (57.5)	0.05
Mobile phone	993	584, (58.8)	
Reading Newspaper	372	204, (54.8)	
Sweeping Grain	98	69, (70.4)	
Other	56	37, (66.1)	
Total Responses	2785	1622, (58.2)	

There was an increasing trend of uptake according to the rating of experience by the participants.

Uptake was found to be highest in those who rated the experience as excellent, followed by those who rated it as very good or good (60.9%, 59.4% and 55.2% respectively, *p* = 0.05). There was no response mentioned as ‘not good’ in the study.

**Fig. 4:**
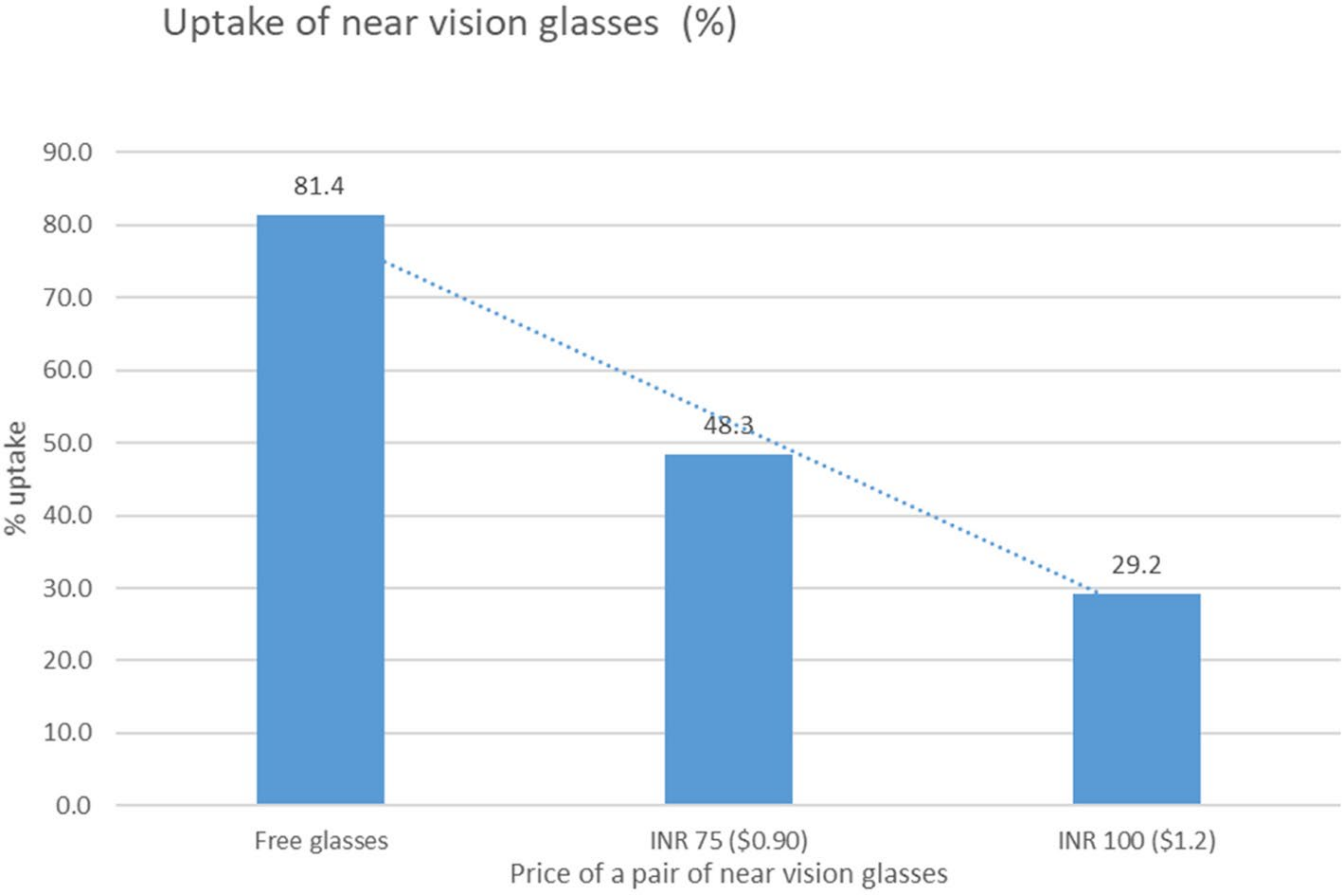
Uptake of glasses related to price

We compared uptake in the three phases (Fig. 4.)

Out of 2790 participants referred by the programme and included in the analysis, a total of 1627 (58.3%) presbyopic individuals had reported at the vision centres to collect glasses. Phase-wise uptake rates were 81.4% (835/1026; 95% CI: 79.0%-83.8%), 48.3% (699/1446; 95% CI: 45.8%—50.9%) and 29.2% (93/318; 95% CI: 24.2%—34.2%), in phases 1,2 and 3 respectively.

Uptake in phase 1, when the glasses were provided free, was found to be significantly greater compared to phases 2 and 3 combined, when glasses were provided at a cost (*p* < 0.001). Uptake in the phase 2, with price at US$0.90, was significantly greater as compared to uptake in phase 3 at US$1.20 (*p* < 0.05). Furthermore, uptake in phase 3 at US$1.20 was significantly lower than when the glasses were provided either free of charge or at US$0.90 in phases 1 and 2 (*p* < 0.001). These comparisons are reported in Table 2.

**Table 2:** Comparison of uptake with glasses provided free vs at a price and comparison of uptake with glasses at two price points

Comparison of uptake with free and any payment	Referred to vision centre	Uptake at vision centre *n*, (%)	p-value
Price- US$0.9 or US$1.2	1764	792, (44.9)	< 0.001
Free	1026	835, (81.4)	
Comparison of uptake at US$1.20 and free or US$0.90			
Free or at US$0.90	2472	1534, (62.1)	< 0.001
Price-US$1.20	318	93, (29.2)	

Uptake of glasses was 57.4% among women and 59.4% among men in all phases combined. This difference was not statistically significant. Among those who use their own transport, 57.4% of the individuals referred procured glasses at the vision centres as compared to 59.2% among those who used public transport. This difference was not statistically significant.

**Fig. 5:**
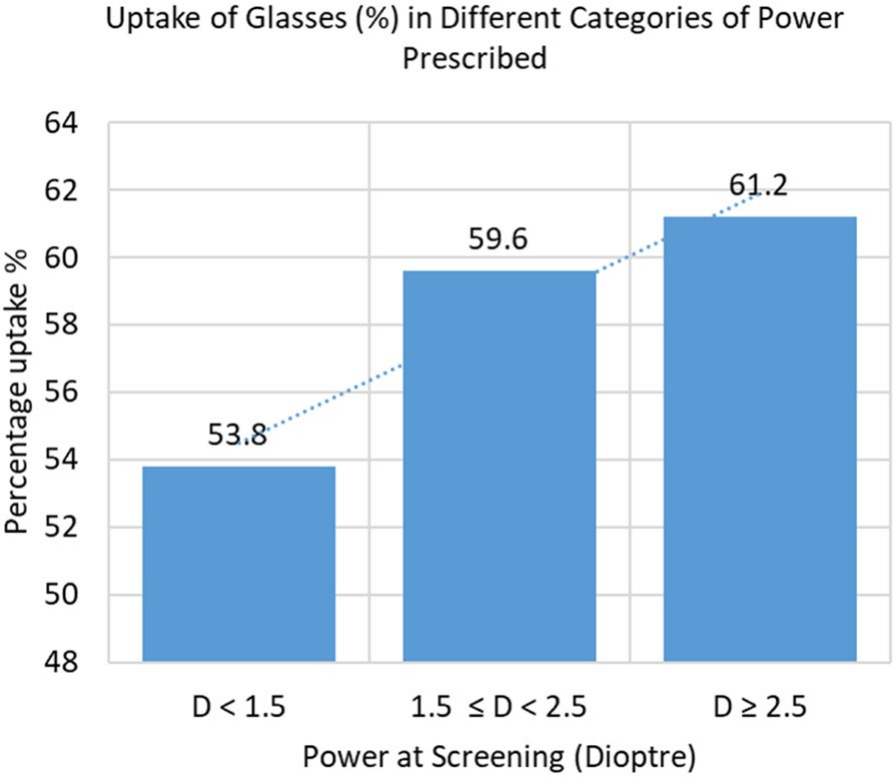
Uptake of glasses related to the power of glasses prescribed

Uptake of glasses among people prescribed glasses of + 2.5D or higher was 61.2% (323/528). This value was greater than that for those prescribed powers between + 1.5D and + 2.5D (900/1511) which was higher than in those prescribed less than + 1.5D (404/751); (Fig. 5). This difference was statistically significant (*p* = 0.01).

We ran multivariable binary logistic regression to identify the factors that were independently associated with improved uptake. These are presented in Table 3.

**Table 3:** Adjusted odds ratios for the associations between various factors and the uptake of near-vision glasses derived from a multivariable binary logistic regression model, with the uptake of near-vision glasses as the outcome variable

	OR	95% CI LL	95% CI UL	*p*-value
Discount 50% (Ref: Discount 30%)	2.77	2.07	3.72	0.000
Free (Ref: Discount 30%)	3.21	2.52	4.11	0.000
Age (continuous)	0.98	0.96	0.99	0.004
Village to vision centre distance (continuous)	0.96	0.93	1.00	0.070
Lens Power at screening (continuous)	1.33	1.08	1.64	0.007
Activity Experience very good or excellent (Ref: Good)	1.42	1.18	1.69	0.000
Transportation facility (Ref: mode of transport not available)	0.91	0.77	1.08	0.281
Sex (Ref: Male)	0.95	0.80	1.12	0.538

Compared with the price of US$1.20, there were 3.2 times greater odds of people procuring glasses if they were given free and 2.8 times greater odds when they were provided glasses at US$0.90. This difference was found to be statistically significant. The odds of uptake increased as the power of the glasses required increased. Those who reported experience as very good or excellent had 1.4 times higher odds of uptake than those who reported the experience as good. The odds of the uptake of glasses decreased with increasing age with age treated as a continuous variable. However, the odds ratio was found to be very close to 1. Although the odds of uptake decreased as the distance of the village from vision centre increased, this difference was not statistically significant. No difference in uptake was found among men and women or among those with or without the availability of mode of transport in their household.

## Discussion

This work was carried out to explore whether the provision of the first pair of near-vision glasses can generate a willingness to acquire the second pair. In this study, the experience of using near-vision glasses at home for half an hour for near activities chosen by the participants was used as a proxy for using the first pair of glasses. In our study, the activity most preferred for receiving experience was threading a needle and using that needle for stitching, followed by using a mobile phone. There were more women in our sample and, overall, a younger presbyopic age group.

We found very high (81%) uptake when glasses were provided free of cost at the vision centre. This study answered our initial research question, which explored the barriers of distance, time, and the indirect cost of travel and time away from home. This percentage uptake was much higher than the uptake recorded in our ongoing program (31%), where individuals are referred for near-vision glasses without providing any ‘experience’. This was also significantly greater than the uptake when glasses were offered at a cost. From a program perspective, this implies a continuous cost of near-vision.

glasses but only a one-time cost of case finding and distribution. If large-scale programmes are planned for the unmet need for presbyopia to improve effective refractive error coverage (eREC), this could imply considerable cost savings as case findings and distribution costs can be substantial in programmes for uncorrected refractive errors [23].

In this study, we went a step further to assess the effect on the barrier of direct cost after being provided a near-vision correction experience (as a proxy for the first pair of glasses). We found that uptake was sensitive to the price. Uptake was significantly greater (48%) at US$0.90 than at US$1.20 (29% uptake). Uptake of 48 percent at a price of US$0.90 was much higher than 31 percent in our ongoing program without any experience provided before referral. However, at a price of US$1.20, even after having had the experience of using near vision correction did not improve the uptake beyond our ongoing program results. These results also match the results of various studies reporting that cost is one of the barriers to the uptake of near-vision glasses [8–14].

In a study by Guan et.al, it was reported that the children who were given vouchers for a free first pair of glasses and asked to travel to a vision centre had significantly higher chances of procuring next pair of glasses, not given free, as compared to children just provided with a prescription after being screened [27]. Thus, highlighting the long-term impact of initial subsidy.

In our study, we found that there was a trend toward decreasing uptake with increasing age, independent of the price point. Although this trend was not very significant, it could be important when high-volume programs for the provision of near-vision glasses are rolled out. Not having someone to accompany was reported as the second most common person-related barrier to uptake of eye care services by people over 40 years of age, in a study in southern India [24]. Thus, models for providing near-vision glasses closer to residences should be explored. A study in an urban environment in Australia revealed better uptake of generalized services than of tailored services in a healthy workplace program [25]. Providing near-vision glasses alongside general health check-ups could be explored.

It was also found that the uptake was greater for those who needed more power from glasses and those who appreciated the experience provided. Both of these factors are related to low priority and lack of felt need generally reported as barriers for the uptake of near-vision glasses [11–14]. This implies that programs planned should have a protocol for the cut-off of power being dispensed, and if beneficiaries are encouraged to use the first pair of near-vision glasses for their favoured activities, there is a greater chance of them acquiring the subsequent pair.

Although it was not statistically significant, increasing distance was associated with a decreasing trend of uptake. Distance has been reported as a barrier to other healthcare interventions, and physical access to quality care has been mentioned as a major issue in a policy paper on healthcare and equity in India [26]. This also suggests that the availability of glasses needs to be ensured closer to the people. While primary eye care centres are obvious options, other models need to be explored to be able to meet unmet needs. Providing near-vision glasses at a reasonable cost closer to the villages has been attempted earlier, including innovative modes such as caravans for the distribution of glasses [27]. This would also need related enabling regulations.

One limitation of the software used for the screening program is that it does not record the number of individuals who decline to participate. However, among the individuals screened in the program and eligible for the study, we had a very high response rate of approximately 95%. While we randomized the villages to be included in this study, all the participants were from villages. Thus, it would be difficult to extrapolate these results in urban scenarios. Uptake of glasses has been linked to literacy in a previous study [10]. As the literacy level in the rural population in this state is lower than that in the urban population [20], uptake may be different if this work is repeated in the urban context. Since this study was nested in an ongoing screening program, only the villages from the region with active program could be randomized. Although the socio-demographic characteristics of the villages were found to be comparable to those of the other villages in our operational districts [20], the findings may not be generalizable to all villages in India. Further studies in diverse geographical areas are recommended.

One of the limitations of our study is the use of a proxy measure of providing experience at the doorstep instead of the actual usage of the first pair over a longer period, e.g., months or years. This was done to reduce the time required to conduct the study, as replacement of near-vision glasses may not be required for one to two years, and the patterns of uptake are unpredictable and less feasible to track routinely. While we believe that using glasses for a year or more, compared with 30 min of experience in our study, would be a stronger motivator to seek a second pair, a longer duration study is recommended to confirm this. In our study, nearly all eligible individuals from a specific village were screened, and all eligible participants were given the opportunity to try near-vision glasses. As a result, the potential for participants to share their experiences with those who were not given this opportunity was limited. We recommend designing a study where a select group of individuals within a geographically close area are provided with this experience. This would allow us to investigate whether a “tipping point” could be reached, where individuals visit vision centres for reading glasses not because they have personally tried them, but because they have heard about them from others.

## Conclusion

Our results show that providing the experience of using near-vision glasses in a rural population in India can increase the desire to acquire these glasses despite the barriers of distance, time, indirect cost and direct cost to some extent. Providing the first pair of near-vision glasses free of cost in programs to ensure initial experience, with the subsequent pairs available at a subsidized cost for these rural populations, may lead to a long-term impact on the unmet need for near-vision glasses.

## Supplementary Information

Supplementary Material 1.

## Data Availability

The datasets used and/or analysed during the current study are available from the corresponding author on reasonable request.
