# Supplementary material for: Willingness to pay for a second pair of near-vision glasses: a cross-sectional study in a rural North Indian population
Source: BMC Public Health. 2025 Apr 22;25:1495. doi: 10.1186/s12889-025-22278-2 (PMC12013087; doi:10.1186/s12889-025-22278-2)

**Supplementary files**

Photo 1. The most used experience- threading the needle and stitching


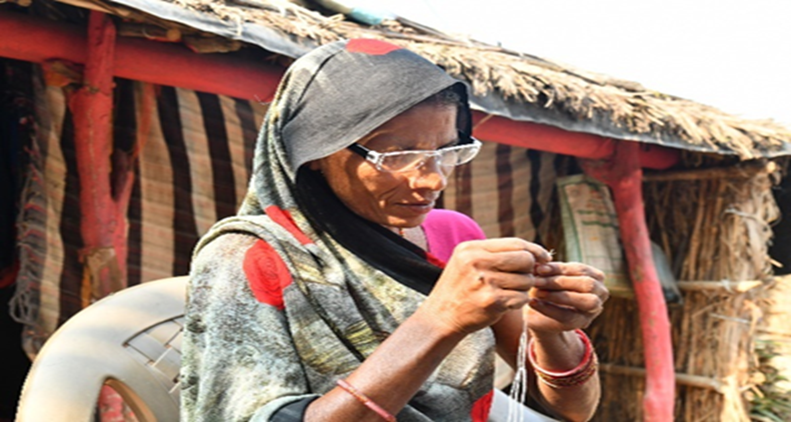


Photo 2. Experience- cleaning the grain


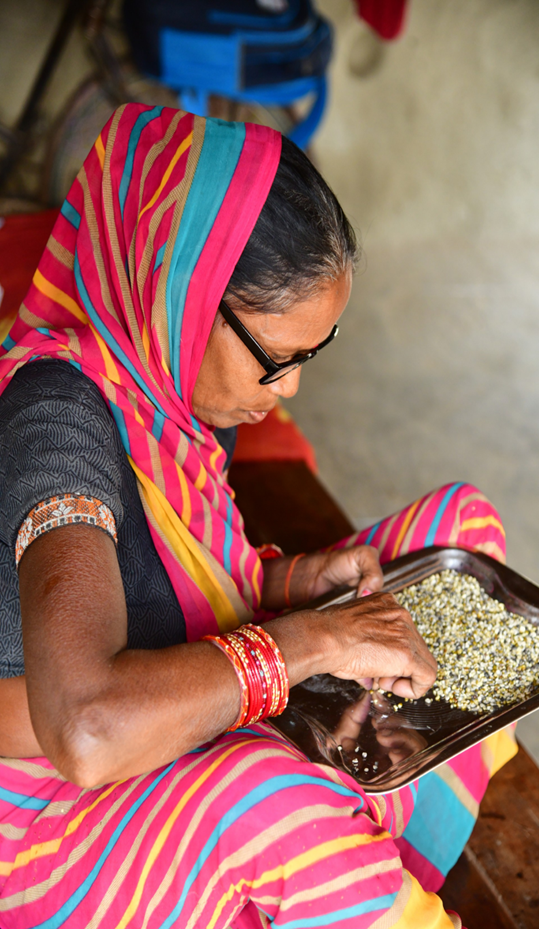


Photo 3: Experience- Using the mobile phone


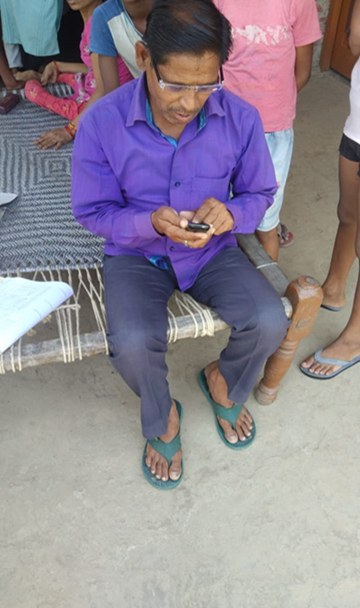

Supplement: Supplementary file 1 — Supplementary Material 1. [file 12889_2025_22278_MOESM1_ESM.docx]
